# Supplementary material for: Effect of Menaquinone-7 Supplementation on Arterial Stiffness in Chronic Hemodialysis Patients: A Multicenter Randomized Controlled Trial
Source: Nutrients. 2023 May 23;15(11):2422. doi: 10.3390/nu15112422 (PMC10255064; doi:10.3390/nu15112422)
Supplement: Supplementary file 1 [file nutrients-15-02422-s001.zip › nutrients-2393290-supplementary.pdf]

Supplementary Table S1. Sensitivity analyses of primary outcomes by adjustment of blood pressures smoking status.

	Control Group	MK-7 Group	Main Model *	Model 2 (Main model + smoking)	Model 3 (Main model + SBP)	Model 4 (Main model + DBP)	Model 5 (Main model + smoking +SBP)	Model 6 (Main model + smoking +DBP)
**Percentage change of cfPWV in 24 weeks (%)**			*p-value*	*p-value*	*p-value*	*p-value*	*p-value*	*p-value*
**All patients (ITT population)**	**N = 44**	**N = 45**						
	-6.7 (-18.8-7.8)	-6.0 (-19.6-2.3)	0.24	0.23	0.23	0.23	0.22	0.22
**Participants with complete 24-week follow-up**	**N = 43**	**N = 43**						
	-6.8 (-19.0, 7.3)	-6.0 (-20.2, 2.3)	0.24	0.24	0.24	0.24	0.24	0.24

* Main model: Multivariable linear mixed effect model (LMM) adjusted by baseline cfPWV with treatment and continuous time interaction-term.
Abbreviation; cfPVW, carotid femoral pulse wave velocity; ITT, intention-to-treat; SBP, systolic blood pressure; DBP, diastolic blood pressure.

Supplementary Table S2. Comparison in change of laboratory parameters at 12 weeks and 24 weeks between treatment and control groups.

Laboratory Parameters	Control Group			TREATMENT GROUP			*p*-Value
	Week 0	Week 12	Week 24	Week 0	Week 12	Week 24	
**BUN preHD** (mg/dL)	55.5 ± 13.7	56.3 ± 16.8	54.7 ± 14.0	59.0 ± 16.5	57.2 ± 13.9	58.3 ± 15.3	0.70
**BUNpostHD** (mg/dL)	10.6 ± 6.8	11.1 ± 5.3	10.4 ± 4.0	10.3 ± 4.2	9.7 ± 4.2	10.6 ± 4.8	0.37
**Serum Cr** (mg/dL)	10.2 ± 2.6	11.4 ± 5.7	10.2 ± 2.6	9.3 ± 2.3	10.5 ± 4.8	9.6 ± 2.4	0.33
**Serum Ca** (mg/dL)	9.3 ± 0.8	9.2 ± 1.2	9.2 ± 0.8	11.2 ± 14.5	9.0 ± 0.9	8.9 ± 1.0	0.44
**Serum PO_4** (mg/dL)	4.3 ± 1.3	4.8 ± 1.7	4.6 ± 1.3	4.2 ± 1.4	4.6 ± 1.2	4.5 ± 1.5	0.70
**Serum Albumin** (mg/dL)	4.3 ± 0.8	4.1 ± 0.6	4.0 ± 0.3	4.1 ± 0.4	4.1 ± 0.3	4.1 ± 0.4	0.03
**FBS** (mg/dL)	97.9 ± 24.8	115.7 ± 33.9	108.7 ± 46.1	115.0 ± 63.2	109.2 ± 25.6	112.2 ± 20.0	0.75
**HbA1C** (%)	5.4 ± 0.8	5.2 ± 0.9	5.0 ± 1.0	5.9 ± 1.3	5.2 ± 0.7	4.9 ± 0.7	0.50
**LDL-C** (mg/dL)	98.6 ± 39.9	89.2 ± 34.0	97.3 ± 31.5	93.2 ± 35.5	90.5 ± 33.7	93.3 ± 33.3	0.98
**iPTH** (pg/mL)	416.6 ± 493.3	663.3 ± 677.1	538.3 ± 579.3	473.0 ± 456.7	715.6 ± 834.8	567.8 ± 765.5	0.58
**Hb** (mg/dL)	10.2 ± 1.7	10.6 ± 2.0	10.3 ± 1.7	10.5 ± 1.3	10.4 ± 1.4	10.7 ± 1.3	0.35

Abbreviation; Ca; calcium, Cr; creatinine, HbA1C; Hemoglobin A1C, HD; hemodialysis; iPTH, intact parathyroid hormone; LDL-C, low density lipoprotein cholesterol, PO_4; phosphate

Supplementary Table S3 Subgroup analyses of DM and non-DM patients in comparison of cfPWV measurement between treatment and control groups at baseline, 12 and 24 weeks

Subgroup DM	Control Group (n=14)	Treatment Group (n=15)	*p*-Value
**cfPWV week 0**	12.9 (11.2, 15.1)	14.7 (12.8, 16.9)	0.07
**Week 12**			
-cfPWV (m/s)	13.1 (11.7, 14.3)	13.1 (11.7, 14.3)	0.68
-Absolute cfPWV change (m/s)	0.4 (-0.9, 1.6)	-1.6 (-2.3, -0.1)	0.06
-Percent change of cfPWV (%)	3.8 (-5.8, 11.6)	-10.0 (-15.9, -0.8)	0.04
-Increased cfPWV from baseline (n/%)	7 (58.3%)	3 (21.4%)	0.05
**Week 24**			
-cfPWV (m/s)	13.1 (12.5, 15.2)	13.9 (11.7, 15.5)	0.81
-Absolute cfPWV change (m/s)	1.3 (0, 2.2)	-0.7 (-2.5, -0.1)	0.01
-Percent change of cfPWV (%)	8.2 (0,17.2)	-5.1 (-16.1, -0.6)	0.01
-Increased cfPWV from baseline (n/%)	8 (72.7%)	3 (21.4%)	0.01
**Subgroup Non-DM**	**Control Group (n = 32)**	**Treatment Group (n = 34)**	***p*-Value**
cfPWV week 0	11.9 (10.8, 13.1)	11.6 (10.6, 12.7)	0.48
**Week 12**			
-cfPWV (m/s)	10.7 (9.1, 12.3)	10.2 (8.2, 12.7)	0.66
-Absolute cfPWV change (m/s)	-1.2 (-2.9, -0.1)	-1.3 (-4.2, -0.2)	0.62
-Percent change of cfPWV (%)	-8.9 (-19.6, -1.0)	-12.0 (-29.8, -1.6)	0.54
-Increased cfPWV from baseline (n/%)	7 (24.1%)	6 (21.4%)	0.81
**Week 24**			
-cfPWV (m/s)	10.4 (9.6, 12.0)	11.0 (9.9, 12.3)	0.66
-Absolute cfPWV change (m/s)	-1.4 (-2.8, -0.1)	-1.1 (-3.0, 0.3)	0.88
-Percent change of cfPWV (%)	-11.1 (-21.7, -1.0)	-8.4 (-21.9, 2.3)	0.86
-Increased cfPWV from baseline (n/%)	9 (28.1%)	10 (34.5%)	0.59

Abbreviation: cfPWV; Carotid-femoral pulse wave velocity, DM; Diabetes mellitus

Supplementary Table S4. Post-hoc univariate and multivariable logistic regression to explore predictors of progression of vascular stiffness.

	**Univariate Analysis**		**Multivariable Analysis**	
**Predictors**	**Unadjusted Odds Ratio (95%CI)**	***p*-Value**	**Adjusted Odds Ratio (95%CI)**	***p*-Value**
MK-7	0.66 (0.27-1.62)	0.37	0.72	0.50
Age (per 1-year increment)	1.03 (0.99-1.07)	0.16	1.02 (0.97-1.07)	0.52
Diabetes	1.74 (0.67-4.53)	0.26	1.21 (0.38-3.80)	0.75
SBP (per 1 mmHg increment)	1.01 (0.99-1.03)	0.34	1.01 (0.98-1.03)	0.65
HD frequency 3 vs. 2/week	0.38 (0.09-1.55)	0.18	0.64 (0.12-3.33)	0.60
Dialysate Ca >=3.0 mmol/L	1.54 (0.63-3.78)	0.34	1.28 (0.49-3.33)	0.61
Current smoking	1.90 (0.11-31.44)	0.66	1.87 (0.09-39.62)	0.09
